# Involvement of TRP Channels in Adipocyte Thermogenesis: An Update

**DOI:** 10.3389/fcell.2021.686173

**Published:** 2021-06-24

**Authors:** Wuping Sun, Yixuan Luo, Fei Zhang, Shuo Tang, Tao Zhu

**Affiliations:** ^1^Department of Pain Medicine and Shenzhen Municipal Key Laboratory for Pain Medicine, Shenzhen Nanshan People’s Hospital and The 6th Affiliated Hospital of Shenzhen University Health Science Center, Shenzhen, China; ^2^Department of Cardiovascular Surgery, Shenzhen Nanshan People’s Hospital and The 6th Affiliated Hospital of Shenzhen University Health Science Center, Shenzhen, China; ^3^Department of Orthopaedics, The Eighth Affiliated Hospital, Sun Yat-sen University, Shenzhen, China; ^4^Department of Respiratory Medicine, Second Affiliated Hospital of Chongqing Medical University, Chongqing, China

**Keywords:** TRP channels, calcium, thermogenesis, energy metabolism, brown adipocytes, beige adipocytes, obesity

## Abstract

Obesity prevalence became a severe global health problem and it is caused by an imbalance between energy intake and expenditure. Brown adipose tissue (BAT) is a major site of mammalian non-shivering thermogenesis or energy dissipation. Thus, modulation of BAT thermogenesis might be a promising application for body weight control and obesity prevention. TRP channels are non-selective calcium-permeable cation channels mainly located on the plasma membrane. As a research focus, TRP channels have been reported to be involved in the thermogenesis of adipose tissue, energy metabolism and body weight regulation. In this review, we will summarize and update the recent progress of the pathological/physiological involvement of TRP channels in adipocyte thermogenesis. Moreover, we will discuss the potential of TRP channels as future therapeutic targets for preventing and combating human obesity and related-metabolic disorders.

## Adipose Tissues and Obesity

Obesity is a severe public health problem causing various diseases including diabetes, hypertension, coronary heart diseases and cancer, which has received considerable attention as a major public health concern (Nguyen and El-Serag, 2010; Blüher, 2019). According to a prediction based on the data from 1975 to 2014 in 200 countries, the prevalence of global obesity will reach to 18% for men and 21% for women by 2025 (NCD Risk Factor Collaboration, 2016). In addition, obesity is becoming prevalent not only in the developed countries, but also in the developing countries (Maharani and Tampubolon, 2016). Therefore, urgent strategies are required for the prevention and reversal of obesity and related metabolic diseases.

Obesity is accompanied by the imbalance of caloric intake and consumption (Hall and Guo, 2017). There is evidence that adipose tissue is involved in the long-term regulation of energy metabolism and fat quality. Adipose tissue is a highly specialized tissue and plays a key role in energy mobilization regulation (Reilly and Saltiel, 2017; Zhai et al., 2020). Two types of adipose tissue have been found in mammals so far, called white adipose tissue (WAT) and brown adipose tissue (BAT) (Cannon and Nedergaard, 2004; Wu et al., 2020). WAT is generally thought as an organ stores excess energy which maintains energy in the form of triglyceride in lipid droplets. However, a new type of brown-like adipocyte was termed beige/brite adipocyte or inducible brown adipocyte has recently been found in human WAT (Sharp et al., 2012; Cypess et al., 2013; Lidell et al., 2013). BAT, which consumes energy and produce heat rapidly, was first discovered in mammalian hibernation research (Ricquier and Kader, 1976). This thermogenic function is mainly mediated by uncoupling protein-1 (UCP1), a polypeptide that exists in the mitochondrial inner membrane of brown adipocytes (Kajimura et al., 2015; Bertholet et al., 2017; Cannon et al., 2020). It has assessed that BAT thermogenesis was decreased in obese mice by oxygen consumption measurement (Martinez-Botas et al., 2000; Ussher et al., 2010). UCP1 expression level in BAT was decreased in almost all obese animals whereas increased in lean animals (Shirkhani et al., 2018). UCP1 knockout (UCP1KO) mice exhibited obesity phenotypes with increased body fat after six months high fat diet (HFD) feeding (Kontani et al., 2005). On the other hand, cold stimulation and/or β3-adrenergic receptor agonist treatment decreased body fat amount by enhancing BAT activity (Lowell and Spiegelman, 2000; Cannon and Nedergaard, 2004). Cold exposure also increased BAT volume and activity, thus increasing energy consumption and promoting weight loss of obese people (Hanssen et al., 2015a, b; Leiria et al., 2019). Several studies have reported that there was a negative correlation between BAT activity/amount and body mass index (BMI) in humans. Imaging data have revealed that patients with higher BMI have lower BAT activity (Cypess et al., 2009; Pfannenberg et al., 2010; Ouellet et al., 2011). Moreover, a single nucleotide substitution at –3826A to G of UCP1 gene polymorphism has been found in human, which decreased the mRNA expression of *Ucp1* and enhanced the age-related obesity and BAT degradation (Nagai et al., 2007; Yoneshiro et al., 2013). Therefore, BAT might play critical role in the regulation of body weight and energy homeostasis.

## Thermogenesis in Brown and Beige Adipocytes

BAT was thought to be restricted only in infants (Lean, 1989; Enerback, 2010). However, previous works have reported that BAT was also found in adult humans by using fluorodeoxyglucose (FDG)-positron emission tomography (PET) in combination with computed tomography (CT) techniques (Cypess et al., 2009; van Marken Lichtenbelt et al., 2009). This novel finding highlights the critical role for BAT in the regulation of energy metabolism and fat deposition (Nedergaard and Cannon, 2010; Nedergaard et al., 2011). Classical brown fat is primarily distributed around interscapular BAT (iBAT), axillary, paravertebral, and perirenal sites (Park et al., 2014). Mitochondria and multilocular lipid droplets were enriched in brown adipocytes, which makes it have remarkable capacity to dissipate energy in the form of heat (Song et al., 2020). UCP1 is expressed in the mitochondria inner membranes of brown adipocytes, which uncouples ATP synthesis from oxidative phosphorylation, thereby dissipating energy as heat. It is well known that BAT non-shivering thermogenesis is controlled directly by sympathetic nervous system innervation and activation. BAT thermogenesis is induced and regulated by the release of norepinephrine from sympathetic nerve terminals and its subsequent binding by β3-adrenergic receptors (Nedergaard et al., 2005; Feldmann et al., 2009). Several studies have shown that how UCP1 is activated, and long chain fatty acid is essential for H^+^ transport (Fedorenko et al., 2012). In addition, another proposed mechanism is that mitochondrial reactive oxygen species (ROS) production regulates UCP1 sulfenylation and thermogenesis (Chouchani et al., 2016). However, signaling pathways for thermogenesis in the downstream of β3-adrenergic receptor activation still have not been well clarified.

Beige adipocyte (UCP1-positive adipocyte) is known to be surrounded by numerous UCP1-negative adipocytes in human WAT (Wu et al., 2012). Beige adipocytes could be recruited after a short-term cold challenge or treatment with β3-adrenergic receptor agonists (Saito et al., 2020). They are very similar to brown adipocytes with high UCP1 expression and thermogenesis (Ye et al., 2013; Li et al., 2014). There are two groups that are a BAT-positive group (subjects have detectable FDG uptake upon cold stimulation) and a BAT-negative group (subjects have undetectable FDG uptake) in humans. Energy metabolism was higher in the BAT-positive group than the BAT-negative group after an acute cold exposure (Orava et al., 2011; Yoneshiro et al., 2011). These studies clearly revealed a critical function for brown and beige adipocytes in cold-induced thermogenesis in humans. Therefore, approaches to modulate brown or beige adipocyte activities might be potential way to prevent and treat human obesity and related metabolic diseases.

## TRP Channels

Transient receptor potential (TRP) ion channels are a major class of calcium-permeable channels, most of which are non-selective cation channels (Montell and Rubin, 1989). TRP channels contain six trans-membrane (TM) domains (TM1–TM6) with a pore loop between TM5 and TM6 (Cao et al., 2013b; Liao et al., 2013; Paulsen et al., 2015; Huynh et al., 2016; Zubcevic et al., 2016). TRP channel superfamily is now subdivided into seven subfamilies and contains 27 channels: TRPV (Vanilloid), TRPC (Canonical), TRPM (Melastatin), TRPML (Mucolipin), TRPN (NomPC), TRPP (Polycystin), and TRPA (Ankyrin) based on their primary amino acid sequences (Ramsey et al., 2006; Wu et al., 2010; Gees et al., 2012). The main signaling pathways in which TRP channels triggered are based on calcium influx through the channels, leading to increases in intracellular Ca^2+^ levels ([Ca^2+^]_*i*_). Numerous studies have shown that some TRP channels are expressed in adipocytes and are involved in energy metabolism and inflammation of adipose tissues, suggesting the potential role of TRP channels in human obesity treatment and prevention (Bishnoi et al., 2018; Uchida et al., 2018; Gao et al., 2019; Zhai et al., 2020). In the present review, we will provide a systematic and brief summary of TRP channels in the regulation of adipocyte thermogenesis and update the recent progress.

## TRPV1

**FIGURE 1:**
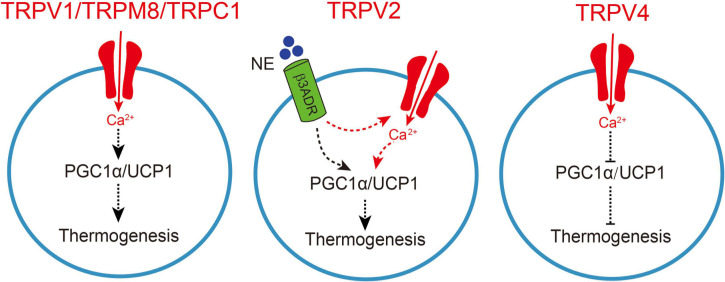
TRP channel-mediated adipocyte thermogenesis. A schematic figure of how TRPV1, TRPV2, TRPV4, TRPM8, and TRPC1-mediated calcium influx regulates thermogenic gene expression in adipocytes, which causes enhanced thermogenesis. Moreover, the increase in sympathetic nerve activity causes norepinephrine release from the sympathetic nerves and activation of β3-adrenergic receptor (β3ADR) in brown adipocytes, TRPV2 synergistically collaborated with β3ADR to involve in the regulation of peroxisome proliferator-activated receptor gamma coactivator-1 a (PGC1a) and uncoupling protein 1 (UCP1), subsequently enhances thermogenesis. On the other hand, TRPV4-mediated calcium influx negatively regulates thermogenic gene expression in adipocytes and subsequently inhibits thermogenesis.

TRPV1 is well-known as a receptor of capsaicin, the pungent ingredient in “hot” chili peppers (Caterina et al., 1997). TRPV1 is activated by a variety of stimuli, including heat (Cao et al., 2013a), protons and capsaicin (Dhaka et al., 2009). In addition, TRPV1 is activated by some compounds in garlic, onion (Salazar et al., 2008), black pepper (Okumura et al., 2010), and other foods, such as gingerol (Iwasaki et al., 2006). TRPV1 has been reported to be expressed in both WAT and BAT (Bishnoi et al., 2013; Kida et al., 2016). TRPV1 expression level is increased in the differentiated HB2 brown adipocytes than in pre-adipocytes (Kida et al., 2016). Moreover, activation of TRPV1 up-regulates the expression of thermogenic genes and induced “browning” in 3T3-L1 adipocytes (Figure 1; Baboota et al., 2014). TRPV1 is expressed in 3T3-L1 pre-adipocytes, adipose tissue of mice and fat tissue of obese humans (Zhang et al., 2007). TRPV1 is activated by dietary capsaicin, a process that induces calcium influx and prevents adipogenesis in 3T3-L1 cells (Zhang et al., 2007) and probably occurs through a calcineurin pathway (Cioffi, 2007). Besides, dietary capsaicin treatment prevented HFD-induced obesity in wild-type (WT) mice *in vivo*, but not in TRPV1KO mice (Zhang et al., 2007; Chen J. et al., 2015; Chen N. et al., 2015). Moreover, TRPV1 was involved in the regulation of energy intake and glucose homeostasis in WAT during HFD-induced obesity (Lee et al., 2015). Absence of TRPV1 exacerbated obese and insulin resistance associated with HFD and aging (Lee et al., 2015). It has also been reported that monoacylglycerol up-regulated UCP1 expression level in brown adipocytes and suppressed accumulation of visceral fat in mice fed with high fat and sucrose through activation of TRPV1 (Iwasaki et al., 2011). Fish oil intake induced UCP1 up-regulation in both brown and white adipose tissues in a TRPV1 dependent manner (Kim et al., 2015; Lund et al., 2018). Oleoylethanolamide, a newly reported TRPV1 ligand, is also involved in the regulation of energy intake and consumption, feeding behavior and weight control (Laleh et al., 2019).

Human studies have showed that capsaicin ingestion enhanced fat oxidation and energy metabolism during aerobic exercise (Shin and Moritani, 2007). A continuous consumption of chili increased the energy metabolism in the middle-aged subjects (Ahuja et al., 2006). Capsinoids, a non-pungent capsaicin analogs, for continuous 1–3 months also increased energy expenditure and fat oxidation with a reduction in abdominal adiposity in overweight and obese subjects (Inoue et al., 2007; Snitker et al., 2009). Moreover, capsaicin and capsinoids as food ingredients enhanced BAT thermogenesis, subsequently decreased fat mass in humans (Yoneshiro et al., 2012; Saito and Yoneshiro, 2013). An epidemiological study suggested that the energy metabolism was enhanced and the prevalence of human obesity in eastern Asian countries was decreased by increasing the consumption of hot foods containing capsaicin (Wahlqvist and Wattanapenpaiboon, 2001).

It has also been reported that capsaicin injection induced adrenaline secretion, this effect was significantly reduced in TRPV1KO mice (Uchida et al., 2017). Capsaicin directly binds to TRPV1 in gastrointestinal tract, produce afferent signal, subsequently transmit to ventromedial hypothalamic nucleus of central nervous system, and finally send signal to WAT. This could promote the expression of β2-adrenoceptor and the production of PRDM16 protein, thus promoting the generation of beige adipocytes, resulting in increased systemic energy expenditure (Ohyama et al., 2016; Saito et al., 2020). Catechins in green tea may activate and recruit BAT by acting on TRPV1/TRPA1 of gastrointestinal sensory neurons in the same way as capsaicin (Mako et al., 2015). Besides, topical application of capsaicin cream in mice resulted in weight loss and adipose tissue weight (Lee et al., 2013). However, whether the regulatory effect of topical capsaicin on obesity is through TRPV1 to activate central nervous system remains to be further studied. These studies clearly demonstrated that targeting TRPV1 and modulation its activity with capsaicin and analogs could be effective approaches for human obesity treatment and prevention, although the anti-obesity effect of TRPV1 activation may be involved not only in adipose tissue, but also in nervous system.

## TRPV2

TRPV2 was initially reported to be activated by noxious heat with an activation temperature threshold of higher than 52°C (Caterina et al., 1999) and found to be activated by several chemicals, e.g., 2-aminoethoxydiphenyl borate (2APB) and lysophosphatidylcholine (LPC) (Juvin et al., 2007; Monet et al., 2009). TRPV2 was also reported to be activated by mechanical stimulation and/or cell swelling (Muraki et al., 2003; Iwata et al., 2009). TRPV2 is expressed in both WAT and BAT (Sun et al., 2017a). TRPV2 is highly expressed in mouse brown adipocytes compared with TRPV1, TRPV3, TRPV4 and TRPM8 (Sun et al., 2016a, b). The expression of TRPV2 was up-regulated at mRNA, protein and functional levels in the differentiated brown adipocytes (Sun et al., 2016b, 2017b). Primary TRPV2-deficient (TRPV2KO) adipocytes show decreased mRNA levels of multiple genes involved in mitochondrial oxidative metabolism, such as *Ucp1* and peroxisome proliferator-activated receptor gamma coactivator 1-alpha (*Pgc1*α). Besides, TRPV2KO adipocytes showed decreased responses to a β-adrenergic receptor agonist, isoproterenol, which might be due to the lack of TRPV2-mediated calcium influx. These results suggested that TRPV2-mediated calcium influx is involved in thermogenic gene induction upon β-adrenergic receptor activation (Figure 1). TRPV2KO mice showed cold intolerance and significantly smaller increases in *Ucp1* mRNA and protein upon cold stimulation at 4°C without changes in their activities. On the other hand, sympathetic nerve activity was not changed in TRPV2KO mice. TRPV2KO mice showed impaired iBAT adaptive thermogenesis upon administration of a β3-adrenergic receptor agonist, BRL37344. Importantly, TRPV2KO mice had significant increases in body weight and adipose tissues upon a HFD treatment (Sun et al., 2016a). Up-regulation of TRPV2 was also observed in obese and diabetic (db/db) mice (Sun et al., 2016a, 2017a). It has also been reported that tart cherry may attenuate adipogenesis by acting directly on the adipose tissue and down-regulating the HFD-induced mRNA expression of TRPV1 and TRPV2 (Cocci et al., 2021). These findings suggested that TRPV2 might be contributed to adipocyte thermogenesis. However, it is necessary to further examine the expression and function of TRPV2 in human BAT and develop specific ligands of TRPV2.

## TRPV3

TRPV3 is a member of the TRPV subfamily which is different from TRPV1 and TRPV2. TRPV3 is well-known to be activated by innocuous temperature around body temperature but initially activated by a high noxious threshold which is over 50°C (Liu and Qin, 2017). The chemical agonists of TRPV3 include camphor, carvacrol, (-)-epicatechin, 2APB, and endogenous ligand farnesyl pyrophosphate (Cheung et al., 2015; Broad et al., 2016). TRPV3 could form heteromeric channels with TRPV1 (Cheng et al., 2012), which also involves in the regulation of adipogenesis and HFD-induced obesity (Cheung et al., 2015). TRPV3 has been reported to be expresses in BAT and WAT (Bishnoi et al., 2013). The expression of TRPV3 was dramatically down-regulated in visceral adipose tissue of obesity mice, including HFD-induced obesity mice, ob/ob and db/db mice (Cheung et al., 2015; Sun et al., 2017a). HFD feeding up-regulated TRPV3 in the medial nucleus tractus solitaries and hypoglossal nucleus, which is accompanied by a reduced expression of proopiomelanocortin and resulted in increased food intake and a gain of body-weight (Hu et al., 2011). Activation of TRPV3 by (-)-epicatechin prevented adipogenesis in 3T3-L1 preadipocytes and played an anti-adipogenic role *in vivo* (Cheung et al., 2015). Besides, berberine alleviates olanzapine-induced obesity by targeting TRPV1/TRPV3 in hypothalamus of mice (Singh et al., 2020). These studies suggested that targeting TRPV3 could be an intriguing approach for the treatment and prevention of obesity. However, the expression of TRPV3 and its role in human obesity needs further exploration.

## TRPV4

TRPV4 was reported to be activated by osmolarity changes or mechanical stimuli (Liedtke et al., 2000; Strotmann et al., 2000; Watanabe et al., 2002a). TRPV4 is also activated by diverse chemical compounds, including a synthetic phorbol ester, 4α-phorbol-12, 13-didecanoate (4α-PDD) and GSK1016790A (Watanabe et al., 2002b; Willette et al., 2008) as well as moderate warmth (temperature threshold higher than 27°C) (Guler et al., 2002; Watanabe et al., 2002b). TRPV4 is expressed in BAT and WAT as well (Sun et al., 2017a, 2020; Uchida et al., 2018). It has been reported that insulin regulates TRPV4-mediated metabolic homeostasis in human white adipocytes (Sanchez et al., 2016). TRPV4 is involved in the modulation of thermogenic and inflammatory pathways in adipose tissue. Knockdown of TRPV4 enhanced the basal and norepinephrine-induced induction of the expression of *Pgc1a* and *Ucp1* (Ye et al., 2012). ERK1/2 were reported to be activated by TRPV4-mediated calcium signaling (Thodeti et al., 2009), and TRPV4 activation-induced calcium influx caused a rapid phosphorylation of ERK1/2 and JNK1/2, which further suppressed the expression of thermogenic genes in 3T3-F442A adipocytes (Figure 1; Ye et al., 2012). Knockdown of TRPV4 also reduced adipose tissue inflammation by inhibiting a number of pro-inflammatory genes (Ye et al., 2012). The expression of TRPV4 in WAT was higher than that in BAT (Sun et al., 2017a). The significant up-regulation of thermogenic gene expression upon TRPV4 inhibition led to the occurrence of WAT “browning” (Ye et al., 2012). TRPV4KO mice exhibited increased muscle energy oxidation and resistance to HFD-induced obese in mice (Kusudo et al., 2012). It has also been reported that treadmill running and rutin ameliorate HFD-induced obesity in mice by suppressing the expression of TRPV4 in adipocytes (Chen N. et al., 2015). Besides, dietary intervention in obese dams protects male offspring from WAT induction of TRPV4, adiposity, and hyperinsulinemia (Janoschek et al., 2016). A human subject-based study has revealed that polymorphisms of TRPV4 gene affects BMI and body fat mass in subjects in Taiwan (Duan et al., 2015). These results revealed an opposite role of TRPV4 in the modulation of adipocyte thermogenesis without knowing the potential mechanisms. Examine the expression and function of TRPV4 in human obesity and developing TRPV4 specific antagonist and *in vivo* examination of the new compounds is warranted.

## TRPM8

The TRPM subfamily consists of eight different subunits, TRPM1 to TRPM8 (Boesmans et al., 2011). TRPM8 is well-known as a menthol receptor which has been reported in the year of 2002 (McKemy et al., 2002). In a human adipocyte cell line, menthol-induced TRPM8 activation increased UCP1 expression, mitochondrial activation and heat production (Figure 1; Rossato et al., 2014). The mRNA and protein expression levels of TRPM8 are significantly increased in the differentiated adipocytes, suggesting the importance of TRPM8 for adipocyte thermogenesis (Rossato et al., 2014). In cultured adipocytes, menthol induced an up-regulation of UCP1 expression which may through a protein kinase A pathway, which subsequently increases BAT thermogenesis and WAT “browning” (Ma et al., 2012; Jiang et al., 2017; Sanders et al., 2020). Besides, it has been reported that cold-sensing TRPM8 channel participates in the regulation of clock and clock-controlled genes in BAT thermogenesis (Moraes et al., 2017). Bioavailable menthol induces energy expending phenotype in differentiating adipocytes (Khare et al., 2019). *In vivo* studies have revealed that dietary menthol supplementation dramatically increased the core body temperatures and locomotor activity in WT mice, but not in TRPM8KO and UCP1KO mice. Menthol supplementation in diet alleviated HFD-induced obesity and insulin resistance as well (Ma et al., 2012; Jiang et al., 2017). And the preventive effect of menthol against HFD-induced obesity and related complications probably involve a glucagon mechanism (Khare et al., 2018). These results suggested that activation of TRPM8 could enhance BAT thermogenesis, which paves a new approach for the treatment and prevention of obesity. TRPM8-dependent increase in core body temperature upon a menthol treatment or cold exposure, which may be mediated by a UCP1 up-regulation (Tajino et al., 2011). Intragastric administration of menthol also enhanced BAT thermogenesis *in vivo* (Tajino et al., 2007; Masamoto et al., 2009). In addition, TRPM8 polymorphism has been reported to be closely correlated with metabolic syndrome in Turkish population (Tabur et al., 2015). Topical menthol appears to increase core body temperature and metabolic rate in adults (Valente et al., 2015). In summary, activation of TRPM8 by its ligands, such as menthol and icilin, mimics adipocyte thermogenesis and might constitute a promising approach to prevent overweight and obesity. However, randomized clinical trials of topical menthol in obese patients are necessary.

## TRPA1

TRPA1 was initially reported as a noxious cold-activated channel with a temperature threshold around 17°C (Story et al., 2003), However, later studies have initiated a heated debate over the role of TRPA1 as a cold sensor. But its cold sensitivity has been disputed later, and the contribution of TRPA1 to cold sensing is currently a matter of strong debate (Bautista et al., 2006; Talavera et al., 2020). TRPA1 is potentially activated by several food components, like allyl isothiocyanate, icilin, menthol, cinnamaldehyde and capsinoids (Laursen et al., 2015). TRPA1 is involved in adipocyte thermogenesis and energy metabolism (Watanabe and Terada, 2015). In HFD-induced obesity mice, oral administration of allyl isothiocyanate reduces body weight, accumulation of lipid droplets in the liver, and white adipocyte size (Lo et al., 2018). It has been reported that cinnamaldehyde reduces visceral fat deposition in HFD-treated mice by stimulating BAT between scapulae (Tamura et al., 2012). Cinnamaldehyde activates TRPA1 in mouse gastric epithelial cells and up-regulates fatty acid oxidation-related genes in adipose tissue (Camacho et al., 2015). Oleuropein aglycone, as an agonist of TRPA1 and TRPV1, enhances the expression of UCP1 in BAT and promote fat thermogenesis by promoting the secretion of norepinephrine (Oi-Kano et al., 2016). It has been hypothesized that menthol-induced thermogenesis in adipocyte probably involved a TRPA1 mechanism as well (Sakellariou et al., 2016). Moreover, TRPA1 activation induces adrenaline secretion and prevent fat accumulation and obese in rodents (Watanabe and Terada, 2015). Intravenous injection of AITC induces adrenaline secretion, and adrenaline promotes the thermogenesis of BAT by activating β3-adrenergic receptor (Saito et al., 2020). These studies suggested that TRPA1 regulates heat production of BAT through central nervous system (Zsombok and Derbenev, 2016). Therefore, activation of TRPA1 by its ligands might be a promising approach for human obesity treatment and prevention. However, the anti-obesity mechanism which TRPA1 and its ligands involved need further exploration. Randomized clinical trials of TRPA1 activation in obese patients are warranted as well.

## TRPC1 and TRPC5

TRPC subfamily includes seven members (TRPC1–7). TRPC channels are usually formed by homo- or heteromeric TRPC proteins (Huang et al., 2011). There is no evidence demonstrate TRPC channels have thermosensitive property so far. TRPC1 is highly expressed in adipocyte depots including BAT and that TRPC1-deficient mice are prone to weight gain and manifest reduced metabolic control (Wolfrum et al., 2018). TRPC1 regulates brown adipocyte activity in a PPARγ-dependent manner, suggesting that TRPC1 is a downstream component of a mechanism that translates metabolic or environmental stimuli into output in the form of BAT activity (Figure 1; Wolfrum et al., 2018). However, an opposite observation has been reported that fat mass and fasting glucose concentrations were lower in TRPC1KO mice that were fed a HFD (45% fat) (Krout et al., 2017). Besides, a mechanically activated TRPC1-like current in white adipocytes was observed (El Hachmane and Olofsson, 2018). It has been reported that either knockdown of TRPC1/TRPC5 *in vitro* or conditional knockout of TRPC5 *in vivo* has increased adiponectin generation in mouse (Sukumar et al., 2012). In addition, both exogenous and endogenous pituitary adenylate cyclase activating polypeptides stimulate proopiomelanocortin neurons and increase energy consumption by activating TRPC1 and TRPC5 channels, which suggests that it is possible to promote BAT thermogenesis by activating TRPC1/TRPC5 in central nervous system (Chang et al., 2020). These studies demonstrated the involvement of TRPC1/TRPC5 in the regulation of energy homeostasis. Further examination of the expression of TRPC1/TRPC5 in human adipose tissues and developing TRPC1/TRPC5 specific agonist are needed.

## TRPP

TRPP is a type of non-selective ion channel, which has been proved to be associated with autosomal dominant polycystic kidney (Moran et al., 2004). TRPP has three family members, TRPP2, TRPP3, and TRPP5. TRPP2, also known as PKD2 or polycystin-2, has been reported to be expressed in adipose tissue, and the expression level of TRPP2 in mature adipocytes is higher than in pre-adipocytes (Moran et al., 2004; Sukumar et al., 2012). Knockdown of TRPP3 suppresses the expression of UCP1 and PGC1α, and attenuates the mitochondrial respiration in adipocytes but has not affected adipogenesis (Goralczyk et al., 2017). These results revealed that TRPP3 might be involved in adipocyte thermogenesis. Further analysis of the mechanisms of TRPP channels in adipocyte thermogenesis is necessary.

## Conclusion and Perspectives

In the past decades, TRP channels have been widely studied in adipocyte thermogenesis, adipogenesis, adipose tissue inflammation, and obesity. TRP channels have been demonstrated to play critical roles in the regulation of energy metabolism for the treatment and prevention of human obesity. In the present review, we summarized and updated the recent progress of the involvement of several TRP channels in adipocyte thermogenesis. It’s worth noting that several concerns still need to be further explored. First of all, the underlying mechanisms which TRP channel-mediated in the thermogenesis process of adipocytes are still controversial, which need to be clearly addressed. Secondly, novel specific ligands of TRP channels are warranted to be developed since there is no specific ligands for TRP channels so far. Thirdly, how do TRP channels exert tissue-specific effects in adipose tissues? These issues are warranted to be addressed by further animal and clinical studies in the future. In conclusion, targeting TRP channels could be promising strategies for clinical treatment and prevention of human obesity and related-metabolic diseases.

## Author Contributions

WS, ST, YL, FZ, and TZ were involved in literature collection, summarization, and written the review manuscript. All authors contributed to the article and approved the submitted version.

## Conflict of Interest

The authors declare that the research was conducted in the absence of any commercial or financial relationships that could be construed as a potential conflict of interest.

## Abbreviations

BMI: body mass index
BAT: brown adipose tissue
CT: computed tomography
FDG: fluorodeoxyglucose
HFD: high fat diet
iBAT: interscapular BAT
WAT: white adipose tissue
UCP1: uncoupling protein-1
PET: positron emission tomography
PGC1 α: peroxisome proliferator-activated receptor gamma coactivator 1-alpha
ROS: reactive oxygen species
TM: trans-membrane
TRP channel: Transient receptor potential channel
TRPV: TRP Vanilloid
TRPV2KO: TRPV2-knockout
TRPC: TRP Canonical
TRPM: TRP Melastatin
TRPML: TRP Mucolipin
TRPN: TRP NomPC
TRPP: TRP Polycystin
TRPA: TRP Ankyrin
WT: wild-type
[Ca^2+^]_*i*_: intracellular Ca^2+^ levels
4 α-PDD: 4 α-phorbol -12, 13-didecanoate.

## References

[B1] Ahuja K. D. Robertson I. K. Geraghty D. P. Ball M. J. (2006). Effects of chili consumption on postprandial glucose, insulin, and energy metabolism. *Am. J. Clin. Nutr.* 84 63–69. 16825682 10.1093/ajcn/84.1.63

[B2] Baboota R. K. Singh D. P. Sarma S. M. Kaur J. Sandhir R. Boparai R. K. (2014). Capsaicin induces ”brite” phenotype in differentiating 3T3-L1 preadipocytes. *PLoS One* 9:e103093. 10.1371/journal.pone.0103093 25072597 PMC4114566

[B3] Bautista D. M. Jordt S. E. Nikai T. Tsuruda P. R. Read A. J. Poblete J. (2006). TRPA1 mediates the inflammatory actions of environmental irritants and proalgesic agents. *Cell* 124 1269–1282. 10.1016/j.cell.2006.02.023 16564016

[B4] Bertholet A. M. Kazak L. Chouchani E. T. Bogaczynska M. G. Paranjpe I. Wainwright G. L. (2017). Mitochondrial Patch Clamp of Beige Adipocytes Reveals UCP1-Positive and UCP1-Negative Cells Both Exhibiting Futile Creatine Cycling. *Cell Metab.* 25 811–822e814. 10.1016/j.cmet.2017.03.002 28380374 PMC5448977

[B5] Bishnoi M. Khare P. Brown L. Panchal S. K. (2018). Transient receptor potential (TRP) channels: a metabolic TR(i)P to obesity prevention and therapy. *Obes. Rev.* 19 1269–1292. 10.1111/obr.12703 29797770

[B6] Bishnoi M. Kondepudi K. K. Gupta A. Karmase A. Boparai R. K. (2013). Expression of multiple Transient Receptor Potential channel genes in murine 3T3-L1 cell lines and adipose tissue. *Pharmacol. Rep.* 65 751–755. 10.1016/s1734-1140(13)71055-7 23950600

[B7] Blüher M. (2019). Obesity: global epidemiology and pathogenesis. *Nat. Rev. Endocrinol.* 15 288–298. 30814686 10.1038/s41574-019-0176-8

[B8] Boesmans W. Owsianik G. Tack J. Voets T. Vanden Berghe P. (2011). TRP channels in neurogastroenterology: opportunities for therapeutic intervention. *Br. J. Pharmacol.* 162 18–37. 10.1111/j.1476-5381.2010.01009.x 20804496 PMC3012403

[B9] Broad L. M. Mogg A. J. Eberle E. Tolley M. Li D. L. Knopp K. L. (2016). TRPV3 in Drug Development. *Pharmaceuticals* 9:h9030055. 10.3390/ph9030055 27618069 PMC5039508

[B10] Camacho S. Michlig S. de Senarclens-Bezençon C. Meylan J. Meystre J. Pezzoli M. (2015). Anti-Obesity and Anti-Hyperglycemic Effects of Cinnamaldehyde via altered Ghrelin Secretion and Functional impact on Food Intake and Gastric Emptying. *Sci. Rep.* 5:7919. 25605129 10.1038/srep07919 PMC4300502

[B11] Cannon B. Nedergaard J. (2004). Brown adipose tissue: function and physiological significance. *Physiol. Rev.* 84 277–359. 10.1152/physrev.00015.2003 14715917

[B12] Cannon B. de Jong J. M. A. Fischer A. W. Nedergaard J. Petrovic N. (2020). Human brown adipose tissue: Classical brown rather than brite/beige? *Exp. Physiol.* 105 1191–1200. 10.1113/EP087875 32378255

[B13] Cao E. Cordero-Morales, Julio F. Liu B. Qin F. Julius D. (2013a). TRPV1 channels are intrinsically heat sensitive and negatively regulated by phosphoinositide lipids. *Neuron* 77 667–679. 23439120 10.1016/j.neuron.2012.12.016 PMC3583019

[B14] Cao E. Liao M. Cheng Y. Julius D. (2013b). TRPV1 structures in distinct conformations reveal activation mechanisms. *Nature* 504 113–118. 10.1038/nature12823 24305161 PMC4023639

[B15] Caterina M. J. Rosen T. A. Tominaga M. Brake A. J. Julius D. (1999). A capsaicin-receptor homologue with a high threshold for noxious heat. *Nature* 398 436–441. 10.1038/18906 10201375

[B16] Caterina M. J. Schumacher M. A. Tominaga M. Rosen T. A. Levine J. D. Julius D. (1997). The capsaicin receptor: a heat-activated ion channel in the pain pathway. *Nature* 389 816–824. 10.1038/39807 9349813

[B17] Chang R. Hernandez J. Gastelum C. Guadagno K. Wagner E. J. (2020). Pituitary Adenylate Cyclase Activating Polypeptide Excites Proopiomelanocortin Neurons: Implications for the Regulation of Energy Homeostasis. *Neuroendocrinology* 111 45–69. 32028278 10.1159/000506367

[B18] Chen J. Li L. Li Y. Liang X. Sun Q. Yu H. (2015). Activation of TRPV1 channel by dietary capsaicin improves visceral fat remodeling through connexin43-mediated Ca2+ influx. *Cardiovasc. Diabetol.* 14:22. 10.1186/s12933-015-0183-6 25849380 PMC4340344

[B19] Chen N. Cheng J. Zhou L. Lei T. Chen L. Shen Q. (2015). Effects of treadmill running and rutin on lipolytic signaling pathways and TRPV4 protein expression in the adipose tissue of diet-induced obese mice. *J. Physiol. Biochem.* 71 733–742. 10.1007/s13105-015-0437-5 26424736

[B20] Cheng W. Yang F. Liu S. Colton C. K. Wang C. Cui Y. (2012). Heteromeric heat-sensitive transient receptor potential channels exhibit distinct temperature and chemical response. *J. Biol. Chem.* 287 7279–7288. 10.1074/jbc.M111.305045 22184123 PMC3293533

[B21] Cheung S. Y. Huang Y. Kwan H. Y. Chung H. Y. Yao X. (2015). Activation of transient receptor potential vanilloid 3 channel (TRPV3) suppresses adipogenesis. *Endocrinology* 156 2074–2086. 10.1210/en.2014-1831 25774551

[B22] Chouchani E. T. Kazak L. Jedrychowski M. P. Lu G. Z. Erickson B. K. Szpyt J. (2016). Mitochondrial ROS regulate thermogenic energy expenditure and sulfenylation of UCP1. *Nature* 532 112–116. 10.1038/nature17399 27027295 PMC5549630

[B23] Cioffi D. L. (2007). The skinny on TRPV1. *Circ. Res.* 100 934–936. 10.1161/01.RES.0000265139.10277.62 17431193

[B24] Cocci P. Moruzzi M. Martinelli I. Maggi F. Micioni Di Bonaventura M. V. (2021). Tart cherry (Prunus cerasus L.) dietary supplement modulates visceral adipose tissue CB1 mRNA levels along with other adipogenesis-related genes in rat models of diet-induced obesity. *Eur. J. Nutr.* 2021:2459–y. 10.1007/s00394-020-02459-y 33386893

[B25] Cypess A. M. Lehman S. Williams G. Tal I. Rodman D. Goldfine A. B. (2009). Identification and importance of brown adipose tissue in adult humans. *N. Engl. J. Med.* 360 1509–1517. 10.1056/NEJMoa0810780 19357406 PMC2859951

[B26] Cypess A. M. White A. P. Vernochet C. Schulz T. J. Xue R. Sass C. A. (2013). Anatomical localization, gene expression profiling and functional characterization of adult human neck brown fat. *Nat. Med.* 19 635–639. 10.1038/nm.3112 23603815 PMC3650129

[B27] Dhaka A. Uzzell V. Dubin A. E. Mathur J. Petrus M. Bandell M. (2009). TRPV1 is activated by both acidic and basic pH. *J. Neurosci.* 29 153–158. 19129393 10.1523/JNEUROSCI.4901-08.2009 PMC2729567

[B28] Duan D. M. Wu S. Hsu L. A. Teng M. S. Lin J. F. Sun Y. C. (2015). Associations between TRPV4 genotypes and body mass index in Taiwanese subjects. *Mol. Genet. Genomics* 290 1357–1365. 10.1007/s00438-015-0996-8 25647731

[B29] El Hachmane M. F. Olofsson C. S. (2018). A mechanically activated TRPC1-like current in white adipocytes. *Biochem. Biophys. Res. Commun.* 498 736–742. 10.1016/j.bbrc.2018.03.050 29524421

[B30] Enerback S. (2010). Human brown adipose tissue. *Cell Metab.* 11 248–252. 10.1016/j.cmet.2010.03.008 20374955

[B31] Fedorenko A. Lishko P. V. Kirichok Y. (2012). Mechanism of fatty-acid-dependent UCP1 uncoupling in brown fat mitochondria. *Cell* 151 400–413. 10.1016/j.cell.2012.09.010 23063128 PMC3782081

[B32] Feldmann H. M. Golozoubova V. Cannon B. Nedergaard J. (2009). UCP1 ablation induces obesity and abolishes diet-induced thermogenesis in mice exempt from thermal stress by living at thermoneutrality. *Cell Metab.* 9 203–209. 10.1016/j.cmet.2008.12.014 19187776

[B33] Gao P. Yan Z. Zhu Z. (2019). The role of adipose TRP channels in the pathogenesis of obesity. *J. Cell. Physiol.* 234 12483–12497. 30618095 10.1002/jcp.28106 PMC13484151

[B34] Gees M. Owsianik G. Nilius B. Voets T. (2012). TRP channels. *Compr. Physiol.* 2 563–608. 10.1002/cphy.c110026 23728980

[B35] Goralczyk A. van Vijven M. Koch M. Badowski C. Yassin M. S. Toh S. A. (2017). TRP channels in brown and white adipogenesis from human progenitors: new therapeutic targets and the caveats associated with the common antibiotic, streptomycin. *FASEB J.* 31 3251–3266. 10.1096/fj.201601081RR 28416581

[B36] Guler A. D. Lee H. Iida T. Shimizu I. Tominaga M. Caterina M. (2002). Heat-evoked activation of the ion channel, TRPV4. *J. Neurosci.* 22 6408–6414. 12151520 10.1523/JNEUROSCI.22-15-06408.2002 PMC6758176

[B37] Hall K. D. Guo J. (2017). Obesity Energetics: Body Weight Regulation and the Effects of Diet Composition. *Gastroenterology* 152:1718. 28193517 10.1053/j.gastro.2017.01.052 PMC5568065

[B38] Hanssen M. J. W. Hoeks J. Brans B. van der Lans A. A. J. J. Schaart G. van den Driessche J. J. (2015a). Short-term cold acclimation improves insulin sensitivity in patients with type 2 diabetes mellitus. *Nat. Med.* 21 863–865. 26147760 10.1038/nm.3891

[B39] Hanssen M. J. W. Lans A. A. J. J. V. D. Brans B. Hoeks J. Jardon K. M. C. Schaart G. (2015b). Short-term Cold Acclimation Recruits Brown Adipose Tissue in Obese Humans. *Diabetes* 65 1179–1189. 26718499 10.2337/db15-1372

[B40] Hu J. Choo H. J. Ma S. X. (2011). Infrared heat treatment reduces food intake and modifies expressions of TRPV3-POMC in the dorsal medulla of obesity prone rats. *Int. J. Hyperthermia* 27 708–716. 10.3109/02656736.2011.601283 21967110 PMC3583363

[B41] Huang J. Du W. Yao H. Wang Y. (2011). “TRPC Channels in Neuronal Survival,” in *TRP Channels*, ed. Zhu M. X. (Boca Raton, FL: CRC Press). 22593969

[B42] Huynh K. W. Cohen M. R. Jiang J. Samanta A. Lodowski D. T. Zhou Z. H. (2016). Structure of the full-length TRPV2 channel by cryo-EM. *Nat. Commun.* 7:11130. 10.1038/ncomms11130 27021073 PMC4820614

[B43] Inoue N. Matsunaga Y. Satoh H. Takahashi M. (2007). Enhanced energy expenditure and fat oxidation in humans with high BMI scores by the ingestion of novel and non-pungent capsaicin analogues (capsinoids). *Biosci. Biotechnol. Biochem.* 71 380–389. 10.1271/bbb.60341 17284861

[B44] Iwasaki Y. Morita A. Iwasawa T. Kobata K. Sekiwa Y. Morimitsu Y. (2006). A nonpungent component of steamed ginger–[10]-shogaol–increases adrenaline secretion via the activation of TRPV1. *Nutrit. Neurosci.* 9 169–178. 17176640 10.1080/110284150600955164

[B45] Iwasaki Y. Tamura Y. Inayoshi K. Narukawa M. Kobata K. Chiba H. (2011). TRPV1 agonist monoacylglycerol increases UCP1 content in brown adipose tissue and suppresses accumulation of visceral fat in mice fed a high-fat and high-sucrose diet. *Biosci. Biotechnol. Biochem.* 75 904–909. 10.1271/bbb.100850 21597186

[B46] Iwata Y. Katanosaka Y. Arai Y. Shigekawa M. Wakabayashi S. (2009). Dominant-negative inhibition of Ca2+ influx via TRPV2 ameliorates muscular dystrophy in animal models. *Hum. Mol. Genet.* 18 824–834. 10.1093/hmg/ddn408 19050039

[B47] Janoschek R. Bae-Gartz I. Vohlen C. Alcazar M. A. Dinger K. Appel S. (2016). Dietary intervention in obese dams protects male offspring from WAT induction of TRPV4, adiposity, and hyperinsulinemia. *Obesity* 24 1266–1273. 10.1002/oby.21486 27106804

[B48] Jiang C. Zhai M. Yan D. Li D. Li C. Zhang Y. (2017). Dietary menthol-induced TRPM8 activation enhances WAT “browning” and ameliorates diet-induced obesity. *Oncotarget* 8 75114–75126. 10.18632/oncotarget.20540 29088850 PMC5650405

[B49] Juvin V. Penna A. Chemin J. Lin Y. L. Rassendren F. A. (2007). Pharmacological characterization and molecular determinants of the activation of transient receptor potential V2 channel orthologs by 2-aminoethoxydiphenyl borate. *Mol. Pharmacol.* 72 1258–1268. 10.1124/mol.107.037044 17673572

[B50] Kajimura S. Spiegelman B. M. Seale P. (2015). Brown and Beige Fat: Physiological Roles beyond Heat Generation. *Cell Metab.* 22 546–559. 10.1016/j.cmet.2015.09.007 26445512 PMC4613812

[B51] Khare P. Chauhan A. Kumar V. Kaur J. Mahajan N. Kumar V. (2019). Bioavailable Menthol (Transient Receptor Potential Melastatin-8 Agonist) Induces Energy Expending Phenotype in Differentiating Adipocytes. *Cells* 8:cells8050383. 10.3390/cells8050383 31027377 PMC6562930

[B52] Khare P. Mangal P. Baboota R. K. Jagtap S. Kumar V. Singh D. P. (2018). Involvement of Glucagon in Preventive Effect of Menthol Against High Fat Diet Induced Obesity in Mice. *Front. Pharmacol.* 9:1244. 10.3389/fphar.2018.01244 30505271 PMC6250823

[B53] Kida R. Yoshida H. Murakami M. Shirai M. Hashimoto O. Kawada T. (2016). Direct action of capsaicin in brown adipogenesis and activation of brown adipocytes. *Cell Biochem. Funct.* 34 34–41. 10.1002/cbf.3162 26781688

[B54] Kim M. Goto T. Yu R. Uchida K. Tominaga M. Kano Y. (2015). Fish oil intake induces UCP1 upregulation in brown and white adipose tissue via the sympathetic nervous system. *Sci. Rep.* 5:18013. 10.1038/srep18013 26673120 PMC4682086

[B55] Kontani Y. Wang Y. Kimura K. Inokuma K. I. Saito M. Suzuki-Miura T. (2005). UCP1 deficiency increases susceptibility to diet-induced obesity with age. *Aging Cell* 4 147–155. 10.1111/j.1474-9726.2005.00157.x 15924571

[B56] Krout D. Schaar A. Sun Y. Sukumaran P. Roemmich J. N. Singh B. B. (2017). The TRPC1 Ca(2+)-permeable channel inhibits exercise-induced protection against high-fat diet-induced obesity and type II diabetes. *J. Biol. Chem.* 292 20799–20807. 10.1074/jbc.M117.809954 29074621 PMC5733613

[B57] Kusudo T. Wang Z. Mizuno A. Suzuki M. Yamashita H. (2012). TRPV4 deficiency increases skeletal muscle metabolic capacity and resistance against diet-induced obesity. *J. Appl. Physiol.* 112 1223–1232. 10.1152/japplphysiol.01070.2011 22207724

[B58] Laleh P. Yaser K. Alireza O. (2019). Oleoylethanolamide: A novel pharmaceutical agent in the management of obesity-an updated review. *J. Cell. Physiol.* 234 7893–7902. 30537148 10.1002/jcp.27913

[B59] Laursen W. J. Anderson E. O. Hoffstaetter L. J. Bagriantsev S. N. Gracheva E. O. (2015). Species-specific temperature sensitivity of TRPA1. *Temperature* 2 214–226. 10.1080/23328940.2014.1000702 27227025 PMC4843866

[B60] Lean M. E. (1989). Brown adipose tissue in humans. *Proc. Nutr. Soc.* 48 243–256. 10.1079/pns19890036 2678120

[B61] Lee E. Jung D. Y. Kim J. H. Patel P. R. Hu X. Lee Y. (2015). Transient receptor potential vanilloid type-1 channel regulates diet-induced obesity, insulin resistance, and leptin resistance. *FASEB J.* 29 3182–3192. 10.1096/fj.14-268300 25888600 PMC4511197

[B62] Lee G. R. Shin M. K. Yoon D. J. Kim A. R. Yu R. Park N. H. (2013). Topical application of capsaicin reduces visceral adipose fat by affecting adipokine levels in high-fat diet-induced obese mice. *Obesity* 21 115–122. 23505175 10.1002/oby.20246

[B63] Leiria L. O. Wang C. H. Lynes M. D. Yang K. Tseng Y. H. (2019). 12-Lipoxygenase Regulates Cold Adaptation and Glucose Metabolism by Producing the Omega-3 Lipid 12-HEPE from Brown Fat. *Cell Metabol.* 30 768.e–783.e. 10.1016/j.cmet.2019.07.001 PMC6774888 31353262

[B64] Li Y. Fromme T. Schweizer S. Schottl T. Klingenspor M. (2014). Taking control over intracellular fatty acid levels is essential for the analysis of thermogenic function in cultured primary brown and brite/beige adipocytes. *EMBO Rep.* 15 1069–1076. 10.15252/embr.201438775 25135951 PMC4253847

[B65] Liao M. Cao E. Julius D. Cheng Y. (2013). Structure of the TRPV1 ion channel determined by electron cryo-microscopy. *Nature* 504 107–112. 10.1038/nature12822 24305160 PMC4078027

[B66] Lidell M. E. Betz M. J. Dahlqvist Leinhard O. Heglind M. Elander L. Slawik M. (2013). Evidence for two types of brown adipose tissue in humans. *Nat. Med.* 19 631–634. 10.1038/nm.3017 23603813

[B67] Liedtke W. Choe Y. Marti-Renom M. A. Bell A. M. Denis C. S. Sali A. (2000). Vanilloid receptor-related osmotically activated channel (VR-OAC), a candidate vertebrate osmoreceptor. *Cell* 103 525–535. 11081638 10.1016/s0092-8674(00)00143-4 PMC2211528

[B68] Liu B. Qin F. (2017). Single-residue molecular switch for high-temperature dependence of vanilloid receptor TRPV3. *Proc. Natl. Acad. Sci. U S A.* 114 1589–1594. 10.1073/pnas.1615304114 28154143 PMC5321025

[B69] Lo C. W. Chen C. S. Chen Y. C. Hsu Y. A. Huang C. C. Chang C. Y. (2018). Allyl Isothiocyanate Ameliorates Obesity by Inhibiting Galectin-12. *Mol. Nutr. Food Res.* 62:e1700616. 10.1002/mnfr.201700616 29345776

[B70] Lowell B. B. Spiegelman B. M. (2000). Towards a molecular understanding of adaptive thermogenesis. *Nature* 404 652–660. 10.1038/35007527 10766252

[B71] Lund J. Larsen L. H. Lauritzen L. (2018). Fish oil as a potential activator of brown and beige fat thermogenesis. *Adipocyte* 7 88–95. 10.1080/21623945.2018.1442980 29521565 PMC6152508

[B72] Ma S. Yu H. Zhao Z. Luo Z. Chen J. Ni Y. (2012). Activation of the cold-sensing TRPM8 channel triggers UCP1-dependent thermogenesis and prevents obesity. *J. Mol. Cell Biol.* 4 88–96. 10.1093/jmcb/mjs001 22241835

[B73] Maharani A. Tampubolon G. (2016). National Economic Development Status May Affect the Association between Central Adiposity and Cognition in Older Adults. *PLoS One* 11:e0148406. 10.1371/journal.pone.0148406 26863443 PMC4749166

[B74] Mako K. Yasushi K. Katsuhiro N. Michihiro T. Yoshihiro K. Osamu S. (2015). Auto-oxidation Products of Epigallocatechin Gallate Activate TRPA1 and TRPV1 in Sensory Neurons. *Chem. Senses* 2015 27–46. 10.1093/chemse/bju057 25422365

[B75] Martinez-Botas J. Anderson J. B. Tessier D. Lapillonne A. Chang B. H. Quast M. J. (2000). Absence of perilipin results in leanness and reverses obesity in Lepr(db/db) mice. *Nat. Genet.* 26 474–479. 10.1038/82630 11101849

[B76] Masamoto Y. Kawabata F. Fushiki T. (2009). Intragastric administration of TRPV1, TRPV3, TRPM8, and TRPA1 agonists modulates autonomic thermoregulation in different manners in mice. *Biosci. Biotechnol. Biochem.* 73 1021–1027. 10.1271/bbb.80796 19420725

[B77] McKemy D. D. Neuhausser W. M. Julius D. (2002). Identification of a cold receptor reveals a general role for TRP channels in thermosensation. *Nature* 416 52–58. 10.1038/nature719 11882888

[B78] Monet M. Gkika D. Lehen’kyi V. Pourtier A. Vanden Abeele F. Bidaux G. (2009). Lysophospholipids stimulate prostate cancer cell migration via TRPV2 channel activation. *Biochim. Biophys. Acta* 1793 528–539. 10.1016/j.bbamcr.2009.01.003 19321128

[B79] Montell C. Rubin G. M. (1989). Molecular characterization of the Drosophila trp locus: a putative integral membrane protein required for phototransduction. *Neuron* 2 1313–1323. 2516726 10.1016/0896-6273(89)90069-x

[B80] Moraes M. N. de Assis L. V. M. Henriques F. D. S. Batista M. L. Jr. Guler A. D. Castrucci A. M. L. (2017). Cold-sensing TRPM8 channel participates in circadian control of the brown adipose tissue. *Biochim. Biophys. Acta Mol. Cell Res.* 1864 2415–2427. 10.1016/j.bbamcr.2017.09.011 28943398

[B81] Moran M. M. Xu H. Clapham D. E. (2004). TRP ion channels in the nervous system. *Curr. Opin. Neurobiol.* 14 362–369. 10.1016/j.conb.2004.05.003 15194117

[B82] Muraki K. Iwata Y. Katanosaka Y. Ito T. Ohya S. Shigekawa M. (2003). TRPV2 is a component of osmotically sensitive cation channels in murine aortic myocytes. *Circ. Res.* 93 829–838. 10.1161/01.RES.0000097263.10220.0C 14512441

[B83] Nagai N. Sakane N. Fujishita A. Fujiwara R. Kimura T. Kotani K. (2007). The -3826 A –> G variant of the uncoupling protein-1 gene diminishes thermogenesis during acute cold exposure in healthy children. *Obes. Res. Clin. Pract.* 1 I–II. 10.1016/j.orcp.2007.02.001 24351450

[B84] NCD Risk Factor Collaboration (2016). Trends in adult body-mass index in 200 countries from 1975 to 2014: a pooled analysis of 1698 population-based measurement studies with 19.2 million participants. *Lancet* 387 1377–1396. 10.1016/S0140-6736(16)30054-X 27115820 PMC7615134

[B85] Nedergaard J. Cannon B. (2010). The changed metabolic world with human brown adipose tissue: therapeutic visions. *Cell Metab.* 11 268–272. 10.1016/j.cmet.2010.03.007 20374959

[B86] Nedergaard J. Bengtsson T. Cannon B. (2011). New powers of brown fat: fighting the metabolic syndrome. *Cell Metab.* 13 238–240. 10.1016/j.cmet.2011.02.009 21356513

[B87] Nedergaard J. Ricquier D. Kozak L. P. (2005). Uncoupling proteins: current status and therapeutic prospects. *EMBO Rep.* 6 917–921. 10.1038/sj.embor.7400532 16179945 PMC1369193

[B88] Nguyen D. M. El-Serag H. B. (2010). The epidemiology of obesity. *Gastroenterol. Clin. North Am.* 39 1–7. 10.1016/j.gtc.2009.12.014 20202574 PMC2833287

[B89] Ohyama K. Nogusa Y. Shinoda K. Suzuki K. Bannai M. Kajimura S. (2016). A Synergistic Antiobesity Effect by a Combination of Capsinoids and Cold Temperature Through Promoting Beige Adipocyte Biogenesis. *Diabetes* 65 1410–1423. 26936964 10.2337/db15-0662 PMC4839206

[B90] Oi-Kano Y. Iwasaki Y. Nakamura T. Watanabe T. Goto T. Kawada T. (2016). Oleuropein aglycone enhances UCP1 expression in brown adipose tissue in high-fat-diet-induced obese rats by activating β-adrenergic signaling. *J. Nutrit. Biochem.* 40 209–218. 27951473 10.1016/j.jnutbio.2016.11.009

[B91] Okumura Y. Narukawa M. Iwasaki Y. Ishikawa A. Matsuda H. Yoshikawa M. (2010). Activation of TRPV1 and TRPA1 by Black Pepper Components. *Biosci. Biotechnol. Biochem.* 74 1068–1072. 20460725 10.1271/bbb.90964

[B92] Orava J. Nuutila P. Lidell M. E. Oikonen V. Noponen T. Viljanen T. (2011). Different metabolic responses of human brown adipose tissue to activation by cold and insulin. *Cell Metab.* 14 272–279. 10.1016/j.cmet.2011.06.012 21803297

[B93] Ouellet V. Routhier-Labadie A. Bellemare W. Lakhal-Chaieb L. Turcotte E. Carpentier A. C. (2011). Outdoor temperature, age, sex, body mass index, and diabetic status determine the prevalence, mass, and glucose-uptake activity of 18F-FDG-detected BAT in humans. *J. Clin. Endocrinol. Metab.* 96 192–199. 10.1210/jc.2010-0989 20943785

[B94] Park A. Kim W. K. Bae K. H. (2014). Distinction of white, beige and brown adipocytes derived from mesenchymal stem cells. *World J. Stem Cells* 2014:33. 10.4252/wjsc.v6.i1.33 PMC3927012 24567786

[B95] Paulsen C. E. Armache J. P. Gao Y. Cheng Y. Julius D. (2015). Structure of the TRPA1 ion channel suggests regulatory mechanisms. *Nature* 520 511–517. 10.1038/nature14367 25855297 PMC4409540

[B96] Pfannenberg C. Werner M. K. Ripkens S. Stef I. Deckert A. Schmadl M. (2010). Impact of age on the relationships of brown adipose tissue with sex and adiposity in humans. *Diabetes* 59 1789–1793. 10.2337/db10-0004 20357363 PMC2889780

[B97] Ramsey I. S. Delling M. Clapham D. E. (2006). An introduction to TRP channels. *Annu. Rev. Physiol.* 68 619–647. 10.1146/annurev.physiol.68.040204.100431 16460286

[B98] Reilly S. M. Saltiel A. R. (2017). Adapting to obesity with adipose tissue inflammation. *Nat. Rev. Endocrinol.* 13 633–643. 10.1038/nrendo.2017.90 28799554

[B99] Ricquier D. Kader J. C. (1976). Mitochondrial protein alteration in active brown fat: A sodium dodecyl sulfate-polyacrylamide gel electrophoretic study. *Biochem. Biophys. Res. Commun.* 73 577–583. 1008874 10.1016/0006-291x(76)90849-4

[B100] Rossato M. Granzotto M. Macchi V. Porzionato A. Petrelli L. Calcagno A. (2014). Human white adipocytes express the cold receptor TRPM8 which activation induces UCP1 expression, mitochondrial activation and heat production. *Mol. Cell Endocrinol.* 383 137–146. 10.1016/j.mce.2013.12.005 24342393

[B101] Saito M. Yoneshiro T. (2013). Capsinoids and related food ingredients activating brown fat thermogenesis and reducing body fat in humans. *Curr. Opin. Lipidol.* 24 71–77. 10.1097/MOL.0b013e32835a4f40 23298960

[B102] Saito M. Matsushita M. Yoneshiro T. Okamatsu-Ogura Y. (2020). Brown Adipose Tissue, Diet-Induced Thermogenesis, and Thermogenic Food Ingredients: From Mice to Men. *Front. Endocrinol.* 11:222. 10.3389/fendo.2020.00222 32373072 PMC7186310

[B103] Sakellariou P. Valente A. Carrillo A. E. Metsios G. S. Nadolnik L. Jamurtas A. Z. (2016). Chronic l-menthol-induced browning of white adipose tissue hypothesis: A putative therapeutic regime for combating obesity and improving metabolic health. *Med. Hypotheses* 93 21–26. 10.1016/j.mehy.2016.05.006 27372851

[B104] Salazar H. Llorente I. Jara-Oseguera A. García-Villegas R. Rosenbaum T. (2008). A single N-terminal cysteine in TRPV1 determines activation by pungent compounds from onion and garlic. *Nat. Neurosci.* 11 255–261. 18297068 10.1038/nn2056 PMC4370189

[B105] Sanchez J. C. Rivera R. A. Munoz L. V. (2016). TRPV4 Channels in Human White Adipocytes: Electrophysiological Characterization and Regulation by Insulin. *J. Cell Physiol.* 231 954–963. 10.1002/jcp.25187 26381274

[B106] Sanders O. D. Rajagopal J. A. Rajagopal L. (2020). Menthol to Induce Non-shivering Thermogenesis via TRPM8/PKA Signaling for Treatment of Obesity. *J. Obes. Metab. Syndr.* 2020:jomes20038. 10.7570/jomes20038 33071240 PMC8017329

[B107] Sharp L. Z. Shinoda K. Ohno H. Scheel D. W. Tomoda E. Ruiz L. (2012). Human BAT possesses molecular signatures that resemble beige/brite cells. *PLoS One* 7:e49452. 10.1371/journal.pone.0049452 23166672 PMC3500293

[B108] Shin K. O. Moritani T. (2007). Alterations of autonomic nervous activity and energy metabolism by capsaicin ingestion during aerobic exercise in healthy men. *J. Nutr. Sci. Vitaminol.* 53 124–132. 17615999 10.3177/jnsv.53.124

[B109] Shirkhani S. Marandi S. M. Kazeminasab F. Esmaeili M. Ghaedi K. Esfarjani F. (2018). Comparative studies on the effects of high-fat diet, endurance training and obesity on Ucp1 expression in male C57BL/6 mice. *Gene* 676 16–21. 10.1016/j.gene.2018.07.015 30201103

[B110] Singh R. Bansal Y. Sodhi R. K. Singh D. P. Kuhad A. (2020). Berberine attenuated olanzapine-induced metabolic alterations in mice: Targeting transient receptor potential vanilloid type 1 and 3 channels. *Life Sci.* 247:117442. 32081663 10.1016/j.lfs.2020.117442

[B111] Snitker S. Fujishima Y. Shen H. Ott S. Pi-Sunyer X. Furuhata Y. (2009). Effects of novel capsinoid treatment on fatness and energy metabolism in humans: possible pharmacogenetic implications. *Am. J. Clin. Nutr.* 89 45–50. 10.3945/ajcn.2008.26561 19056576 PMC3151435

[B112] Song A. Dai W. Jang M. J. Medrano L. Li Z. Zhao H. (2020). Low- and high-thermogenic brown adipocyte subpopulations coexist in murine adipose tissue. *J. Clin. Invest.* 130 247–257. 10.1172/JCI129167 31573981 PMC6934193

[B113] Story G. M. Peier A. M. Reeve A. J. Eid S. R. Mosbacher J. Hricik T. R. (2003). ANKTM1, a TRP-like channel expressed in nociceptive neurons, is activated by cold temperatures. *Cell* 112 819–829. 10.1016/s0092-8674(03)00158-2 12654248

[B114] Strotmann R. Harteneck C. Nunnenmacher K. Schultz G. Plant T. D. (2000). OTRPC4, a nonselective cation channel that confers sensitivity to extracellular osmolarity. *Nat. Cell Biol.* 2 695–702. 10.1038/35036318 11025659

[B115] Sukumar P. Sedo A. Li J. Wilson L. A. O’Regan D. Lippiat J. D. (2012). Constitutively active TRPC channels of adipocytes confer a mechanism for sensing dietary fatty acids and regulating adiponectin. *Circ. Res.* 111 191–200. 10.1161/CIRCRESAHA.112.270751 22668831 PMC3605801

[B116] Sun W. Li C. Zhang Y. Jiang C. Zhai M. Zhou Q. (2017a). Gene expression changes of thermo-sensitive transient receptor potential channels in obese mice. *Cell Biol. Int.* 41 908–913. 10.1002/cbin.10783 28464448

[B117] Sun W. Uchida K. Tominaga M. (2017b). TRPV2 regulates BAT thermogenesis and differentiation. *Channels* 11 94–96. 10.1080/19336950.2016.1228401 27573536 PMC5398572

[B118] Sun W. Uchida K. Suzuki Y. Zhou Y. Kim M. Takayama Y. (2016a). Lack of TRPV2 impairs thermogenesis in mouse brown adipose tissue. *EMBO Rep.* 17 383–399. 10.15252/embr.201540819 26882545 PMC4772987

[B119] Sun W. Uchida K. Takahashi N. Iwata Y. Wakabayashi S. Goto T. (2016b). Activation of TRPV2 negatively regulates the differentiation of mouse brown adipocytes. *Pflugers Arch.* 468 1527–1540. 10.1007/s00424-016-1846-1 27318696

[B120] Sun W. Yu Z. Yang S. Jiang C. Kou Y. Xiao L. (2020). A Transcriptomic Analysis Reveals Novel Patterns of Gene Expression During 3T3-L1 Adipocyte Differentiation. *Front. Mol. Biosci.* 7:564339. 10.3389/fmolb.2020.564339 33195411 PMC7525235

[B121] Tabur S. Oztuzcu S. Duzen I. V. Eraydin A. Eroglu S. Ozkaya M. (2015). Role of the transient receptor potential (TRP) channel gene expressions and TRP melastatin (TRPM) channel gene polymorphisms in obesity-related metabolic syndrome. *Eur. Rev. Med. Pharmacol. Sci.* 19 1388–1397. 25967713

[B122] Tajino K. Hosokawa H. Maegawa S. Matsumura K. Dhaka A. Kobayashi S. (2011). Cooling-sensitive TRPM8 is thermostat of skin temperature against cooling. *PLoS One* 6:e17504. 10.1371/journal.pone.0017504 21407809 PMC3047576

[B123] Tajino K. Matsumura K. Kosada K. Shibakusa T. Inoue K. Fushiki T. (2007). Application of menthol to the skin of whole trunk in mice induces autonomic and behavioral heat-gain responses. *Am. J. Physiol. Regul. Integr. Comp. Physiol.* 293 R2128–R2135. 10.1152/ajpregu.00377.2007 17761510

[B124] Talavera K. Startek J. B. Alvarez-Collazo J. Boonen B. Alpizar Y. A. Sanchez A. (2020). Mammalian Transient Receptor Potential TRPA1 Channels: From Structure to Disease. *Physiol. Rev.* 100 725–803. 10.1152/physrev.00005.2019 31670612

[B125] Tamura Y. Iwasaki Y. Narukawa M. Watanabe T. (2012). Ingestion of cinnamaldehyde, a TRPA1 agonist, reduces visceral fats in mice fed a high-fat and high-sucrose diet. *J. Nutrit. Sci. Vitaminol.* 58:9. 23007061 10.3177/jnsv.58.9

[B126] Thodeti C. K. Matthews B. Ravi A. Mammoto A. Ghosh K. Bracha A. L. (2009). TRPV4 channels mediate cyclic strain-induced endothelial cell reorientation through integrin-to-integrin signaling. *Circ. Res.* 104 1123–1130. 10.1161/CIRCRESAHA.108.192930 19359599 PMC2754067

[B127] Uchida K. Dezaki K. Yoneshiro T. Watanabe T. Yamazaki J. Saito M. (2017). Involvement of thermosensitive TRP channels in energy metabolism. *J. Physiol. Sci.* 67 549–560. 10.1007/s12576-017-0552-x 28656459 PMC10717017

[B128] Uchida K. Sun W. Yamazaki J. Tominaga M. (2018). Role of Thermo-Sensitive Transient Receptor Potential Channels in Brown Adipose Tissue. *Biol. Pharm. Bull.* 41 1135–1144. 10.1248/bpb.b18-00063 30068861

[B129] Ussher J. R. Koves T. R. Cadete V. J. Zhang L. Jaswal J. S. Swyrd S. J. (2010). Inhibition of de novo ceramide synthesis reverses diet-induced insulin resistance and enhances whole-body oxygen consumption. *Diabetes* 59 2453–2464. 10.2337/db09-1293 20522596 PMC3279530

[B130] Valente A. Carrillo A. E. Tzatzarakis M. N. Vakonaki E. Tsatsakis A. M. Kenny G. P. (2015). The absorption and metabolism of a single L-menthol oral versus skin administration: Effects on thermogenesis and metabolic rate. *Food Chem. Toxicol.* 86 262–273. 10.1016/j.fct.2015.09.018 26429629

[B131] van Marken Lichtenbelt W. D. Vanhommerig J. W. Smulders N. M. Drossaerts J. M. Kemerink G. J. Bouvy N. D. (2009). Cold-activated brown adipose tissue in healthy men. *N. Engl. J. Med.* 360 1500–1508. 10.1056/NEJMoa0808718 19357405

[B132] Wahlqvist M. L. Wattanapenpaiboon N. (2001). Hot foods–unexpected help with energy balance? *Lancet* 358 348–349. 10.1016/S0140-6736(01)05586-6 11502310

[B133] Watanabe H. Davis J. B. Smart D. Jerman J. C. Smith G. D. Hayes P. (2002a). Activation of TRPV4 channels (hVRL-2/mTRP12) by phorbol derivatives. *J. Biol. Chem.* 277 13569–13577. 10.1074/jbc.M200062200 11827975

[B134] Watanabe H. Vriens J. Suh S. H. Benham C. D. Droogmans G. Nilius B. (2002b). Heat-evoked activation of TRPV4 channels in a HEK293 cell expression system and in native mouse aorta endothelial cells. *J. Biol. Chem.* 277 47044–47051. 10.1074/jbc.M208277200 12354759

[B135] Watanabe T. Terada Y. (2015). Food Compounds Activating Thermosensitive TRP Channels in Asian Herbal and Medicinal Foods. *J. Nutr. Sci. Vitaminol.* 61 (Suppl.) S86–S88. 10.3177/jnsv.61.S86 26598901

[B136] Willette R. N. Bao W. Nerurkar S. Yue T. L. Doe C. P. Stankus G. (2008). Systemic activation of the transient receptor potential vanilloid subtype 4 channel causes endothelial failure and circulatory collapse: Part 2. *J. Pharmacol. Exp. Ther.* 326 443–452. 10.1124/jpet.107.134551 18499744

[B137] Wolfrum C. Kiehlmann E. Pelczar P. (2018). TRPC1 regulates brown adipose tissue activity in a PPARgamma-dependent manner. *Am. J. Physiol. Endocrinol. Metab.* 315 E825–E832. 10.1152/ajpendo.00170.2017 29989850

[B138] Wu J. Bostrom P. Sparks L. M. Ye L. Choi J. H. Giang A. H. (2012). Beige adipocytes are a distinct type of thermogenic fat cell in mouse and human. *Cell* 150 366–376. 10.1016/j.cell.2012.05.016 22796012 PMC3402601

[B139] Wu L. J. Sweet T. B. Clapham D. E. (2010). International Union of Basic and Clinical Pharmacology. LXXVI. Current progress in the mammalian TRP ion channel family. *Pharmacol. Rev.* 62 381–404. 10.1124/pr.110.002725 20716668 PMC2964900

[B140] Wu M. Junker D. Branca R. T. Karampinos D. C. (2020). Magnetic Resonance Imaging Techniques for Brown Adipose Tissue Detection. *Front. Endocrinol.* 11:421. 10.3389/fendo.2020.00421 32849257 PMC7426399

[B141] Ye L. Kleiner S. Wu J. Sah R. Gupta R. K. Banks A. S. (2012). TRPV4 is a regulator of adipose oxidative metabolism, inflammation, and energy homeostasis. *Cell* 151 96–110. 10.1016/j.cell.2012.08.034 23021218 PMC3477522

[B142] Ye L. Wu J. Cohen P. Kazak L. Khandekar M. J. Jedrychowski M. P. (2013). Fat cells directly sense temperature to activate thermogenesis. *Proc. Natl. Acad. Sci. U S A.* 110 12480–12485. 10.1073/pnas.1310261110 23818608 PMC3725077

[B143] Yoneshiro T. Aita S. Kawai Y. Iwanaga T. Saito M. (2012). Nonpungent capsaicin analogs (capsinoids) increase energy expenditure through the activation of brown adipose tissue in humans. *Am. J. Clin. Nutr.* 95 845–850. 10.3945/ajcn.111.018606 22378725

[B144] Yoneshiro T. Aita S. Matsushita M. Kameya T. Nakada K. Kawai Y. (2011). Brown adipose tissue, whole-body energy expenditure, and thermogenesis in healthy adult men. *Obesity* 19 13–16. 10.1038/oby.2010.105 20448535

[B145] Yoneshiro T. Ogawa T. Okamoto N. Matsushita M. Aita S. Kameya T. (2013). Impact of UCP1 and beta3AR gene polymorphisms on age-related changes in brown adipose tissue and adiposity in humans. *Int. J. Obes.* 37 993–998. 10.1038/ijo.2012.161 23032405

[B146] Zhai M. Yang D. Yi W. Sun W. (2020). Involvement of calcium channels in the regulation of adipogenesis. *Adipocyte* 9 132–141. 10.1080/21623945.2020.1738792 32175809 PMC7153653

[B147] Zhang L. L. Yan Liu D. Ma L. Q. Luo Z. D. Cao T. B. Zhong J. (2007). Activation of transient receptor potential vanilloid type-1 channel prevents adipogenesis and obesity. *Circ. Res.* 100 1063–1070. 10.1161/01.RES.0000262653.84850.8b 17347480

[B148] Zsombok A. Derbenev A. (2016). TRP Channels as Therapeutic Targets in Diabetes and Obesity. *Pharmaceuticals* 9:50. 10.3390/ph9030050 PMC5039503 27548188

[B149] Zubcevic L. Herzik M. A. Jr. Chung B. C. Liu Z. Lander G. C. Lee S. Y. (2016). Cryo-electron microscopy structure of the TRPV2 ion channel. *Nat. Struct. Mol. Biol.* 23 180–186. 10.1038/nsmb.3159 26779611 PMC4876856

